# Identification of water use behavior and calculation of water footprint: a case study

**DOI:** 10.1007/s13201-021-01459-5

**Published:** 2021-06-29

**Authors:** Pelin Okutan, Atilla Akkoyunlu

**Affiliations:** Boğaziçi University Civil Engineering Department, 34342 Bebek, Istanbul, Turkey

**Keywords:** Water footprint, Virtual water footprint, Climate change, Sustainability, Resilience, Smart city

## Abstract

This article adds to the literature on the investigation of water use behavior of people under their daily routines. A self-administrated survey of water users was conducted for both graduate and undergraduate students at Boğaziçi University, Turkey in 2019. This study quantifies and maps the water footprint (WF) of Boğaziçi University (BU). It reports preferences of students and personnel in terms of indoor water use, outdoor water use, and virtual water use such as transportation, shopping, and dietary preferences. Water footprints are estimated per person for both engineering students and all students of BU. WF of an average BU student is above the average WF of Turkey as well as the average global WF. The attributes that influence the water use behavior of people were broadly categorized into two groups, which were dietary preferences and shopping preferences. Moreover, the eating pattern of a person regarding whether a person consumes meat was the largest contributor to the WF of BU. The results of this study can help to develop a basic understanding of WF and how it is affected by people's choices. This study is also unique by calculating water footprint in terms of direct and virtual water footprint by considering people’s daily choices in Turkey.

## Introduction

Water is vital for humans since it is used in the production of crops and grains, production of industrial materials as well as everyday materials. Only 2.5% of all the water available in the world is freshwater. Furthermore, of all the freshwater available in the world, 68.7% is in ice and snow form; 29.9% is in groundwater and 0.26% is found in lakes, rivers, and reservoirs (Shiklomanov 2000). The increasing population in the world also pressures natural resources by increasing demand for energy and water. Moreover, the population in Turkey increased from 28 million in the 1960s to 83 million in 2020 (TSI 2021). According to the predictions of Addams et al. (2009), the global annual water requirements in 2030 would be 6900 m^3^ exceeding more than 65% of total accessible and reliable water sources. The total usable freshwater potential of Turkey is around 112 billion m^3^, which makes the available amount of water per capita is around 1350 m^3^. Furthermore, according to the predictions of the Turkish Statistical Institute (TSI), Turkey’s population is predicted to reach approximately 93 million in 2030 (TSI 2018). In this case, the amount of water available per capita per year, which is now 1302 m^3^, will decrease to 1204 m^3^ in 2030 (General Directorate of State Hydraulic Works 2016). Population increase may create environmental issues such as diminishing freshwater supplies. Domestic water use share is around 11% in the world (WWAP 2019), and domestic water use share in Turkey is 16%. Actions should be taken to decrease domestic water use which in return help to save water that is already diminishing.

Studies show that Turkey has one of the highest levels of water security threat of the countries in Europe. It is densely populated, and most areas of the country face high levels of water stress. It is possible to predict the pressures on water resources with the effects of factors such as climate change, the growth rate of population, and change in water consumption behavior. Studies also show that problem is likely to increase with population increase and the potential drying associated with the rising temperatures due to climate change (Ericson et al. 2006; Met Office 2011). According to the Fourth Assessment Report of the Intergovernmental Panel on Climate Change (IPCC 2007), Turkey is located in the Mediterranean Basin that is vulnerable to the adverse impacts of climate change. Moreover, according to Şen (2013), Mediterranean Basin is defined as one of the most vulnerable regions to future climate change and is considered to be affected by the reduction in precipitation the most. Climate change does not occur uniformly throughout the world. Turkey may experience an increase in the arid areas that may possibly lead to increased water stress around the southern Mediterranean areas (Gao and Giorgi 2008; Şen 2013).

The Intergovernmental Panel on Climate Change (IPCC) states that climate change is extremely likely due to human activities. Human activities are hard to be predicted. Therefore, IPCC developed a collection of scenarios to project climate change to decrease the obscurity of human actions. These scenarios include driving forces such as socioeconomic development, technological change, and demographic development (IPCC 2013). According to World Economic Forum Water Initiative (2008), cities consume 60% of all water allocated for human use. In 2009, 44% of the world’s population, which is 2.8 billion people, lived in areas where the supply of water is insufficient. People who will have an insufficient water reach are expected to be around 4 billion by 2030 (World Economic Forum Water Initiative 2008). An inefficient supply of water creates many problems in human health and accordingly in economic growth by increasing food prices for those who even have not enough money to have water. Primarily, one needs to calculate water footprint to understand how much freshwater is consumed, how much freshwater is available, and how they can preserve the water supply.

In order to release the pressure on freshwater resources, seawater can also be used through desalination. However, desalination of seawater is not only costly but also demands too much energy, and people who live on land have limited access to seawater. This study suggests that following smart and green solutions by putting humans in the center of all actions with support by government and agencies will ease the pressure on freshwater resources. Hence, the current water use trends of people should be investigated by calculating their water footprint.

Water footprint term and its calculation are first introduced by Hoekstra (2002). Hoekstra (2002) states that water footprint is necessary to measure and calculate the freshwater use through the supply chain of the processes and products. The water footprint can be calculated in terms of water used in the production process of a product, shipment, and supply chain of the product as a volume of water. After Hoekstra's (2002) introduction of water footprint, water footprint has been used as an indicator of freshwater use of "processes, products, consumers, a group of consumers, a business, a business sector, and humanity" as a whole (Water Footprint Assessment Manual 2011). To sum up, water footprint is the volume of water used directly or indirectly. The direct water footprint is the volume of water used in the form of taking shower, drinking, washing dishes, doing laundry, and washing cars. However, indirect water footprint is the volume of water used in the production, and supply chain of the product. Moreover, virtual water is water consumed by the production phase of everyday materials depending on where they are produced and used, and the production method. Since there is a limited supply of freshwater, water is also treated economically as virtual water trade. Virtual water allows nations to trade from "water-rich" nations to "water-poor" nations.

This study provides insight into the water footprint of a university campus by investigating water use behaviors. This study also aims to provide insight into larger systems since universities are the small representations of cities with their governors, citizen, and facilities. This research contributes to the literature by focusing on the water use behavior of residents of a university campus. It aims to analyze the impacts of daily routines on the water use behavior and water footprint of an ordinary user. The main contribution of this paper is introducing insights into the decisions of the residents of a university campus and how their decisions affect the water use behavior virtually and directly.

## Theory and methods

### Data collection and descriptive statistics

The survey is conducted in Boğaziçi University, which has six campuses located in both the European side and Asian sides of Istanbul. University stretches over a 168-hectare area that hosts Rectorate and Administrative units along with 4 faculties, 2 institutes, and 10 dormitories. This study investigates indoor water use, outdoor water use, and virtual water use of the members of Boğaziçi University. An online questionnaire is distributed randomly to undergraduate and graduate students of Boğaziçi University, Istanbul in 2019.

The questionnaire includes education and accommodation of each individual while stating their indoor water use frequencies, use of energy and water-efficient appliances, and outdoor water use and virtual water use. Individuals were asked about their daily trips, their tendency toward recycling and reuse of materials, source of electricity at their homes, eating habits, and feeding animals.

The questionnaire contains questions about a variety of socioeconomic characteristics such as age, education, accommodation, number of households. Indoor water use behaviors such as average shower time, average faucet running time for both kitchen and bathroom, number of average flushing, dishwashing methods, laundry methods, availability of a greywater system, and availability of low-flow faucets in their living environments are enquired. Besides, for outdoor water use behaviors such as availability and size of lawn or garden at their living environments, availability of plants that require less or no water, availability of rain barrel and swimming pool, duration of keeping the pool covered, frequency of car washing and car washing methods are also enquired. Lastly, virtual water use behaviors such as kilometers traveled per week, source of electricity, frequency of shopping, paper recycling habits, plastic recycling habits, bottle and can recycling habits, donation preferences, dietary preferences, frequency of meat consumption, amount of cat and dog food consumption are examined.

The online questionnaire was distributed randomly to 12,351 undergraduate students and 4986 graduate students of Boğaziçi University. The sample size (*n*) was determined to be a total of 391 people with a 95% confidence level according to the Yamane formula (Yamane 1967) which is used for calculating sample size with 95% confidence level and *P* = 0.5 are assumed for Eq. (1)

1 $$n=\frac{\mathrm{N}}{1+\mathrm{N}.{\mathrm{e}}^{2}}$$

where *n* is the sample size, *N* is the population size and e is the level of precision. The survey was answered by 394 people online, which is larger than the sample size with a 95% confidence level.

Linear regression was performed in IBM SPSS Statistics Data Editor 25. According to the importance predictor, meat consumption nearly determines total WF since it has an importance of 56% on total WF. Also, shopping and cat or dog food spending have importance of 26% and 14% on the total WF, respectively. These attributes will be explained in the following section. Predictor importance test shows how much each content affects total water footprint in percentages. Moreover, the *t*-test is performed to determine the significance of the model. Model is significant (Sig. = 0) with a 95% confidence interval and 393 degrees of freedom (df). Also, the lower confidence interval is 5906.89 liters per capita per day, while the upper is 6257 liters per capita per day.

### Direct water footprint calculation

Direct water footprint depends on water used directly and changes according to the faucet type and the duration of the water use. For example, low-flow faucets do not allow water to flow as much as conventional faucets. For these reasons, faucet types in the shower, bathroom sink, and kitchen; average use time of students; the average bath and average toilet use frequencies; frequency of doing laundry and washing dishes and the cleaning methods are asked. Lawn and garden watering, swimming pool, car washing-related questions are asked to determine outside direct water footprint. The questionnaire contains questions about possessing a garden, dimensions of the possessed; possessing a swimming pool, and time interval of keeping it covered; possessing a car, and washing methods.

Water used directly can be compensated by creating green opportunities to reuse the water. The questionnaire contains questions about green applications such as greywater systems, having a rain barrel, and xeriscaping help to save water and decrease direct water footprint. Greywater systems help to reuse water and decrease the direct water footprint. Greywater systems that are installed at houses allow the household to collect and reuse water from the kitchen, laundry, shower, and bath to water their garden. Xeriscaping is another method of preserving freshwater use since it is the activity of planting in the garden that will decrease evapotranspiration by 33% (Hoekstra et al. 2011a, b). A rain barrel system collects approximately 5000 l of rainwater per year from the outlet of the roof (EPA 2016). Details of direct water footprint calculation are given in the following subsection.

#### Showers

Water use in the shower is directly related to showerhead type. Low-flow showerheads do not allow water to flow as much and fast as conventional showerheads. The flow rate of a low-flow showerhead is 0.1577 l/min and that of a conventional shower head is 0.31545 l/min (Alliance for Water Efficiency 2018a, b, c). For these reasons, showerhead types were asked and each preference was assigned to a flow rate. To determine average water use in the home, the average shower time of students was collected as a time interval and this value was multiplied with the assigned flow rate.

#### Bathtub

The average bath uses 133 l of water (Alliance for Water Efficiency 2018a, b, c). The amount of water use in baths per capita per day was determined by multiplying the frequency of taking bath with the time multiplier.

#### Bathroom sink

Faucet type affects the water use directly. Low-flow faucets allow saving water since they do not flow as much and fast as old showerheads. The flow rate of a low-flow faucet is 5.68 l/min and that of a conventional faucet is 18.97 l/min (Residential End Uses of Water 2018). To determine the average water use in bathroom sinks, the time interval representing the running time of bathroom sinks while brushing teeth or shaving was multiplied with the assigned flow rate.

#### Toilets

People use the toilet from 3 times per day to 3 times per week, which results in 1.7 feces waste per day on average (Mayoclinic 2018). Moreover, people flush 5 times per day on average (Hoekstra et al. 2011a, b). Not flushing every time can save water rather than flushing every time. An average person who does not flush whenever they use the toilet flushes 1.7 times per day, whereas flushing every time is 5 flushes per day, on average (Hoekstra et al. 2011a, b). Low-flow toilets use 5.68 liters per flush, and conventional toilets use 18.97 liters per flush. To determine the average water use in toilets, the flow rate based on toilet type was multiplied with the assigned flushes per day.

#### Kitchen

To determine the water use in the kitchen, the time interval that students leave the kitchen faucet open for rinsing food, cleaning but not washing dishes were multiplied with the assigned flow rate based on faucet type.

#### Washing dishes

The type of dishwashing method affects water use for example hand-wash consumes more water comparing to conventional and energy-efficient dishwashers. Moreover, eating out or using disposable dishes are directly related to the number of people living in the house since one disposable dish requires 19 l of water to be produced. Water use in washing dishes depends on the method people use while washing dishes as well as the number of times dishwashing per time and the number of people residing in the house. To determine the average water use in washing dishes, frequency of dishwashing and dishwashing per time were multiplied with the assigned flow rate of the method whether it is handwashing, using the conventional dishwasher, energy/water-efficient dishwasher, or using disposable dishes or eating out; the result is then divided by the number of people in the house to determine the water use per capita.

#### Laundry

The number of times that students do their laundry affects their water footprint. Conventional washing machines consume more water per load when comparing to energy-efficient washing machines or laundromats. To determine the average water use in laundry, the frequency of doing laundry and laundry per time was multiplied with the assigned flow rate of the method whether using a conventional washer, energy/water-efficient washer or using laundromats; the result is then divided by the number of people in the house to determine the water use per capita.

#### Lawn and garden watering

Garden or lawn affects water footprint. The size of the place that is being watered is directly proportional to water use. The water use in lawn and garden was determined by multiplying the frequency of gardening and gardening per time with assigned water use amount depending on the size of the garden or whether students have a garden; the result is then divided by the number of people in the house to determine the water use per capita.

#### Swimming pool

An average pool requires 70 m^3^ to fill. If the user does not cover it while using, the pool loses approximately 4 m^3^ per month by evaporation (Hoekstra et al. 2011a, b). To determine the water use due to having a swimming pool, the duration of the pool is uncovered was multiplied with evaporation and added the fill amount and then, the result was divided by the number of people in the house to determine the water use per capita.

#### Car washing

Type of washing method and the frequency of washing affect water footprint. The type of car wash such as garden hose, full-service car wash, self-service car wash, or whether having a car determines the amount of water use per wash. To determine the water use in car washing, the amount of water use per wash depending on the wash type was multiplied with the frequency of wash; then, the result was divided by the number of people that use the same car.

#### Greywater

Greywater systems create an opportunity to reuse water and works to decrease the direct water footprint. Greywater systems that are installed at houses allow the household to collect and reuse water from the kitchen, laundry, shower, and bath to water their garden. A typical household holds 56 m^3^ reusable water per year from the greywater system (James 2010). To determine water saved by greywater systems, 56 m^3^ was divided by the total number of people using the same system and by total days per year.

#### Xeriscaping

Xeriscaping is another method of preserving freshwater use since it is the activity of planting in the garden that will decrease evapotranspiration by 33% (Hoekstra et al. 2011a, b). To determine water saved by xeriscaping, the water use in gardening was multiplied by 33%.

#### Rain barrel

A rain barrel system is just a barrel that is connected to the outlet of the roof and collects rainwater in it. A rain barrel system can collect 5000 l of rainwater per year which is approximately 15 liters per day (EPA 2016). To determine water saved by rain barrel, 15 l was divided by the total number of people using the same barrel.

### Indirect water footprint calculation

The indirect water footprint is the water footprint that is not used directly but in the virtual form. The virtual water content of a product is the volume of freshwater used to produce the product, measured at the place where the product was produced. It is the sum of water use in every step of the production chain. Moreover, the other definition of virtual water is the volume of water that would have been required to produce the product at the place where the product is consumed. The real water content of products is negligible if compared to virtual water content because the direct water footprint of a process is the indirect water footprint of the next process. Furthermore, the water footprint is a multidimensional indicator, not only referring to the water volume used, but also making explicit where the water footprint is located, what source of water is used, and when the water is used. The additional information is crucial to assess the impacts of the water footprint of a product.

To make familiar with the concept of virtual water content and indirect water footprint, a pullover can be investigated. A pullover will require cotton to be grown, ginning and spinning of the fibers, weaving, sewing, and wet processing of the fabric to ultimately have the finished product. Each step has a direct water footprint and an indirect water footprint. The direct water footprint of one process becomes the indirect water footprint of the next process. In this way, the full amount of water consumed or polluted is considered in the product's water footprint.

To limit this study, it is assumed that only gasoline use, electricity use and supply methods, shopping preferences, dietary preferences, and buying cat and dog food increase the indirect water footprint and only reusing, donating, recycling, and using renewable resources as an energy source decrease the indirect water footprint. For example, producing gasoline and transporting it to the sink consume water. Therefore, on an average car, driving 1 km consumes 1.72 l of water (King and Webber 2008). The water footprint of electricity depends on the source of energy, operation, and construction phases of the energy platform (Mekonnen et al. 2015). Water is required to make all the things people buy and use in their daily life, including plastics such as toys and food packaging, electronics, furniture, textile, and packaging and shipping for all daily life products.

Dietary preferences constitute a considerable amount of water footprint (Table 1) since consuming 1 kg of beef increases the water footprint by 15,500 l (Hoekstra et al. 2011a, b). The water footprint of beef and grains strongly varies depending on the production region, feed composition, and origin of the feed ingredients. Eating habits can affect the water footprint of each individual for example preferences of vegans do not consume as much water as preferences of meat-eaters. To determine the dietary consumption patterns of vegans and vegetarians, it is assumed that vegans eat no meat or dairy, while vegetarians eat dairy but no meat. In all cases except for vegans, egg consumption holds constant (Hoekstra et al. 2011a, b). Furthermore, owning or feeding a cat or dog also increases water footprint since for every $1 spent on the cat or dog food, 760 l of water are required to produce and transport it (Hoekstra et al. 2011a, b). Details of indirect water footprint calculation are given in the following subsection.

**Table 1:** Global average WF of food items (Hoekstra et al. 2011a, b; Mekonnen et al. 2015)

Food item	Unit	Global average WF (l/kg)
Apple or pear	1 kg	700
Banana	1 kg	860
Beef	1 kg	15,500
Sheep and Goat meat	1 kg	8763
Bread (from wheat)	1 kg	1300
Cabbage	1 kg	200
Cereals	1 kg	1644
Cheese	1 kg	5000
Butter	1 kg	5553
Chicken	1 kg	3900
Chocolate	1 kg	24,000
Cucumber	1 kg	240
Fruits	1 kg	962
Groundnuts (in shell)	1 kg	3100
Lettuce	1 kg	130
Maize	1 kg	900
Olives	1 kg	4400
Orange	1 kg	460
Peach or Nectarine	1 kg	1200
Potato	1 kg	250
Rice	1 kg	3400
Tomato	1 kg	180
Vegetables	1 kg	322
Beer (from barley)	1 glass of 250 ml	75
Milk	1 glass of 250 ml	250
Coffee	1 glass of 125 ml	140
Tea	1 cup of 250 ml	30
Wine	1 glass of 125 ml	120

#### Gasoline

Producing and refining transportation fuels like oil, natural gas, and biofuels require a lot of water. Researchers at the Lawrence Berkeley National Laboratory estimate that the USA withdraws one to two billion gallons of water to refine nearly 800 million gallons of petroleum products like gasoline every day. To complete all the steps required to produce a liter of gasoline takes, on average, 3–6 l of water (Hoekstra et al. 2011a, b).

Producing gasoline and transporting it to the sink consume water. Therefore, on an average car, driving 1 km consumes 1.72 l of water (King and Webber 2008). To determine the water use for driving, the drive distance was multiplied by 1.72 and divided by the total number of people using the same barrel.

#### Electricity WF of Istanbul

The WF of electricity (m^3^/TJ) refers to the volume of water consumed and polluted in the different stages of the supply chain of electricity. Mekonnen et al. (2015) studied the WF of electricity in terms of fuel supply, construction, and operation. The first stage is relevant only for fuel-based electricity (when electricity is based on coal, lignite, oil, gas, uranium, or biomass). In the other cases (hydro, solar, wind, and geo-electricity), they only considered construction and operation stages.

In this study, the electricity WF of Istanbul was calculated. Electricity is supplied from various sources in Istanbul such as solar, wind, biogas, and mostly from natural gas (IBB 2018a, b). Constructing the facility that electricity will be supplied, operating the facility, and supplying the electricity from that facility consume some amount of water. The values were determined as water footprint as m^3^ per terajoule (TJ) of electricity supplied to the city of Istanbul.

89.9% of the electricity of Istanbul is supplied from natural gas, and 1.9% is supplied from biogas. Yet, the water footprint of natural gas is zero, while that of biogas is 28,666 m^3^/d. To determine water use in electricity of Istanbul, the type of supply whether it is natural gas, solar, wind, or biogas was multiplied with the water footprint of that supply and divided by the population of Istanbul. The WF of electricity of Istanbul was calculated as 1.91 l of water per capita per day. This can be published as a different study by authors.

#### Electricity WF of Boğaziçi University

To determine the electricity WF of BU, the type of electricity was asked. The Electricity WF was taken zero if the electricity was supplied from a renewable source such as solar panels and wind, otherwise, it was accepted as 1.91 liters per capita per day.

#### Shopping

Since products consume water in their production and supply processes, water footprint related to the shopping preferences of the students occurs. Water is required to make all the things people buy and use in their daily life, including plastics such as toys and food packaging, electronics, furniture, textile, and packaging and shipping for all daily life products. According to Hoekstra and Chapagain (2004), shopping for only basics consumes 1100 l of water per person per day, whereas shopping too much consumes approximately 4 times of shopping for basics, 4415 liters per capita per day.

To determine the WF of shopping, shopping preferences were asked and assigned as the amount calculated by Hoekstra and Chapagain (2004)*.*

#### Diet

According to Hoekstra and Mekonnen (2011), in an industrial beef production system, it takes on average three years before the animal is slaughtered to produce about 200 kg of boneless beef. The animal consumes nearly 1300 kg of grains (wheat, oats, barley, corn, dry peas, soybean meal, and other small grains), 7200 kg of roughages (pasture, dry hay, silage, and other roughages), 24 cubic meters of water for drinking and 7 cubic meters of water for servicing. This means that to produce one kilogram of boneless beef, 6.5 kg of grain, 36 kg of roughages, and 155 l of water (only for drinking and servicing) are used. Producing the volume of feed requires about 15,300 l of water on average. The water footprint of 1 kg of beef thus adds up to 15,500 l of water. This still excludes the volume of polluted water that may result from the leaching of fertilizers in the feed crop field or from surplus manure reaching the water system. The numbers provided are estimated global averages. Therefore, the water footprint of beef will strongly vary depending on the production region, feed composition, and origin of the feed ingredients.

**Fig. 1:**
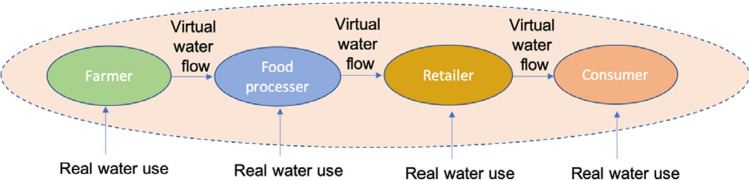
Virtual water chain (Hoekstra and Mekonnen 2011)

The direct water footprint of a previous step is an indirect water footprint of the next step; for example, food is received by the consumer via a retailer, the retailer receives food via food processer, food processer receives food via farmer. Each step uses real water, also during the stages of storage, transportation virtual water flow occurs. This is called virtual water chain and explained by Hoekstra and Mekonnen (2011) (Fig. 1). That is why direct water footprint is negligible compared to indirect water footprint. Furthermore, virtual water footprints of different types of food can be seen in Table 1. Eating habits can affect the water footprint of each individual for example preferences of vegan do not consume as much water as preferences of meat-eaters. To determine the dietary consumption patterns of vegans and vegetarians, it was assumed that vegans eat no meat or dairy, while vegetarians eat dairy but no meat. In all cases except for vegans, egg consumption holds constant (Hoekstra et al. 2011a, b).

#### Cat and dog food

Boğaziçi University is also known for its cats and dogs. Buying cat and dog food also increases the water footprint. For every $1 spent on cat or dog food, 760 l of water are required to produce and transport it (Hoekstra et al. 2011a, b).

#### WF of recycling paper, plastic, cans, and bottles, textile for Turkey

Recycling materials reduce water footprint since recycled materials need less energy and water in the production process rather than raw materials. Amounts of recycled paper, can, bottles, and plastic are found in the Bulletin of Ministry of Environment and Urban for recycled materials in 2016, and the amounts recycled are given in Table 2 (CSB 2018). The amount of textile recycled is 10,000 tons in 2010 (Üçgül and Turak 2015).

**Table 2:** Water footprint (l/c.d.) of recycling and donation

Recycling	Amount recycled (ton/year)	Amount recycled (kg/c.d.)	Water saved (m^3^/kg)	WS (l/c.d.)
Paper	1,199,606	0.041178	26	1.07062
Plastic	498,887	0.017125	185	3.168088
Bottle	231,306	0.00794	90	0.714583
Can	169,798	0.005828	7	0.040799
Textile	10,000	0.000372	170	0.063176

## Results

### Outcomes of the study

Hoekstra founded Water Footprint Network and later developed a methodology in the name of the Water Footprint Assessment to quantify the water use in 2002 (Hoekstra et al. 2011a, b). According to Hoekstra et al. (2011a, b), the water footprint of world average is 3794 liters per capita per day (l/c.d.), whereas the water footprint of Boğaziçi University is 6082 liters per capita per day and the water footprint of engineering students of Boğaziçi University is 6287 liters per capita per day; water footprint of Turkey is 4498 liters per capita per day. The water footprint of Boğaziçi University is higher than that of the world and Turkey.

**Fig. 2:**
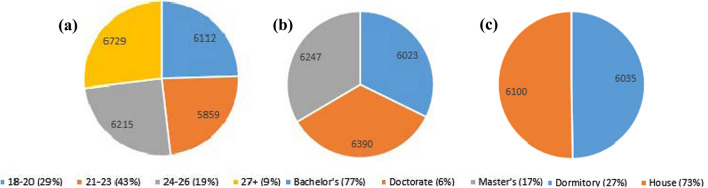
**a** Water Footprint according to age. **b** Water footprint according to degrees. **c** Water Footprint according to accommodation

The questionnaire was answered by 394 students of Boğaziçi University in the 2018/2019 academic year. 9% of total respondents are between 18 and 20 years old, 43% of total respondents are between 21 and 23 years old, 19% of total respondents are between 24 and 26 years old, and lastly, 9% of total respondents are 27 years old and above (Fig. 2a). Water footprints for those age periods are 6112 liters per capita per day, 5859 liters per capita per day, 6215 liters per capita per day, and 6729 liters per capita per day, respectively. 77% of total respondents are bachelor’s students, 17% of that are master’s students, and 6% of total respondents are Ph.D. students. The water footprint of Bachelor’s is 6023 liters per capita per day, while Ph.D. and Master’s students are 6390 and 6247 liters per capita per day, respectively (Fig. 2b).

48% of total respondents are engineering students, and the rest is randomly distributed. 73% of students are staying off-campus, while 27% of students are accommodated in dormitories. Water footprints according to accommodation preferences are 6035 liters per capita per day, and 6100 liters per capita per day, respectively (Fig. 2c).

**Fig. 3:**
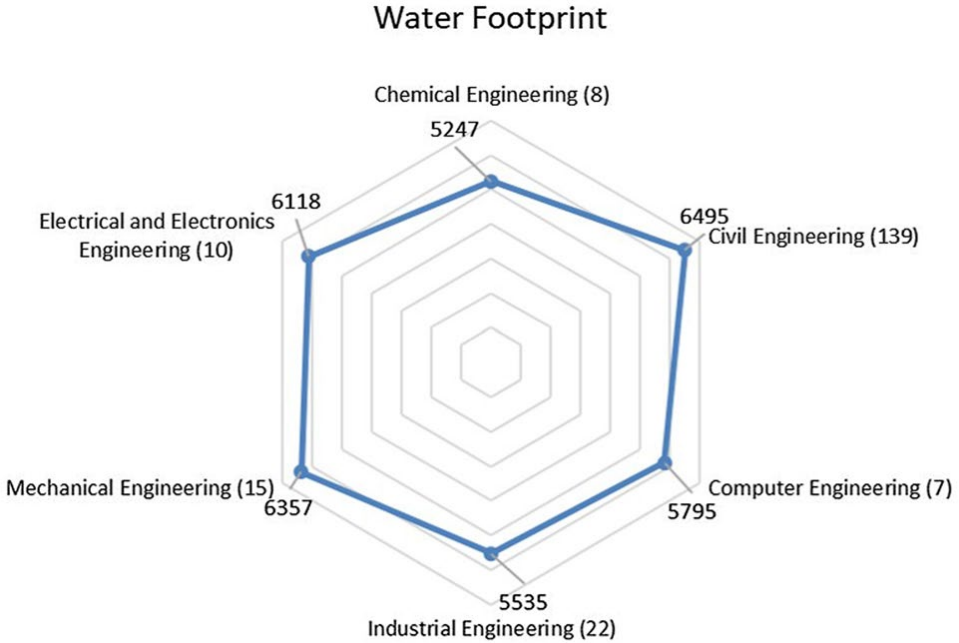
Radar graph representation of WF (l/c.d.) of engineering students

From Fig. 3, it can be seen that 7 Computer Engineering students, 8 Chemical Engineering students, 10 Electrical and Electronic Engineering students, 15 Mechanical Engineering students, 22 Industrial Engineering students, and 139 Civil Engineering students responded to the water footprint survey. The water footprints of engineering students are 5795 liters per capita per day, 5247 liters per capita per day, 6118 liters per capita per day, 6357 liters per capita per day, 5535 liters per capita per day, and 6495 liters per capita per day, respectively.

The direct and indirect water footprints of students of Boğaziçi University are 446 liters per capita per day, 5636 liters per capita per day, respectively. The direct and indirect water footprints of Engineering students of Boğaziçi University are 438 liters per capita per day, 5850 liters per capita per day, respectively. Consequently, the water footprint of students and Engineering students of Boğaziçi University is 6082 liters per capita per day and 6287 liters per capita per day, respectively.

The minimum total WF is 3494 liters per capita per day, the minimum indirect WF is 3526 liters per capita per day, and − 32 liters per capita per day for direct WF. Direct WF is negative because that student has a greywater system in a home which can save 155 l of water per capita per day since the student lives alone. The maximum total WF is 21874 liters per capita per day since the student eats meat in all their meal and does not recycle. Maximum indirect WF is 17708 liters per capita per person, and maximum direct WF is 4166 liters per capita per day due to that student has a garden to water. Median levels of total, indirect and direct WF are 5566, 5199, and 379 liters per capita per day, respectively.

Thirty-three students have a water footprint of fewer than 4498 liters per capita per day which is the water footprint of Turkey. These students, generally, have regular recycling behavior, using energy and water-efficient devices at home. The minimum water footprint is related to have a greywater system installed at home, have a rain barrel system, not having a swimming pool. However, 15 students have a water footprint of more than 10,000 liters per capita per day, which exceeds the world average of 3794 liters per capita per day. These students, in general, are eating meat in every meal, spending more than 200 TRY/month on a cat or dog food, having an irregular recycling behavior, using conventional machines that do not help water and energy efficiency, taking bath every day, not having greywater or rain barrel system.

**Fig. 4:**
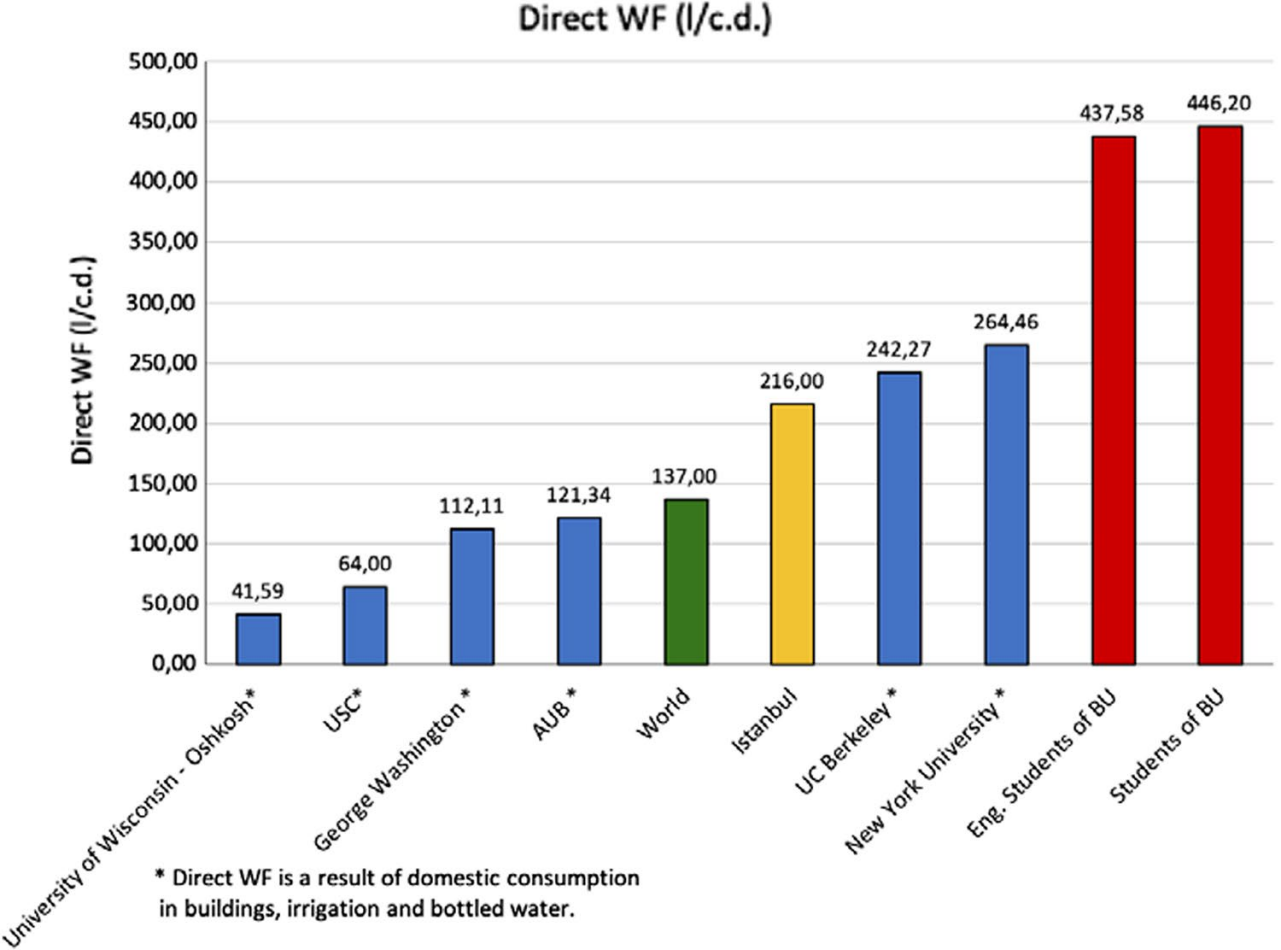
Direct WF of various universities and Total WF of BU

**Fig. 5:**
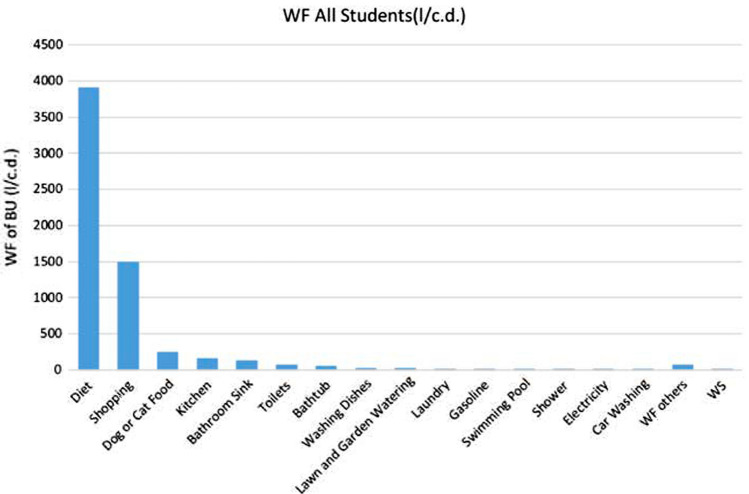
WF (l/c.d.) results of students of BU

The average water footprints of each activity are shown in Table 3. Boğaziçi University is compared with the water footprint of the world per capita per day and that of since there is no total water footprint analysis in the literature that covers both direct and indirect water use of students. Direct water footprint comparison is shown in Fig. 4 where direct water footprints of universities marked with (*) are not as accurate as direct WF of BU. This is because those universities only used domestic consumption in the buildings and irrigation in the universities with bottled water use (AUB 2015). Figure 5 shows the distribution of the water footprint attributes; dietary preferences, shopping preferences, and cat/dog food use are the highest contributors of the calculated WF.

**Table 3:** WF results for students of Boğaziçi University and engineering students

Type	WF all students (l/c.d.)	WF engineering students (l/c.d.)
*Indoor direct use*		
Shower	2.92	2.69
Bathtub	50.7	53.45
Bathroom sink	125.26	118.75
Toilets	64.84	65.77
Kitchen	154.38	156.6
Washing dishes	21.5	20.07
Laundry	11.51	10.73
Greywater*	6.2	6.04
*Outdoor direct use*		
Lawn and garden watering	14.81	7.83
Xeriscaping*	0.99	1.63
Rain barrel*	0.45	0.35
Swimming pool	6.46	7.82
Car washing	1.44	1.93
*Virtual water use*		
Gasoline	6.94	8.13
Electricity	1.8	1.82
Shopping	1489	1448
Recycling paper*	0.52	0.52
Recycling plastic*	1.33	1.35
Recycling cans and bottles*	0.33	0.32
Donating textile*	0.056	0.05
Diet	3901	4168
Dog or cat food	240	226
Total	6082	6287

(*) Represents saved water because of the activity

**Fig. 6:**
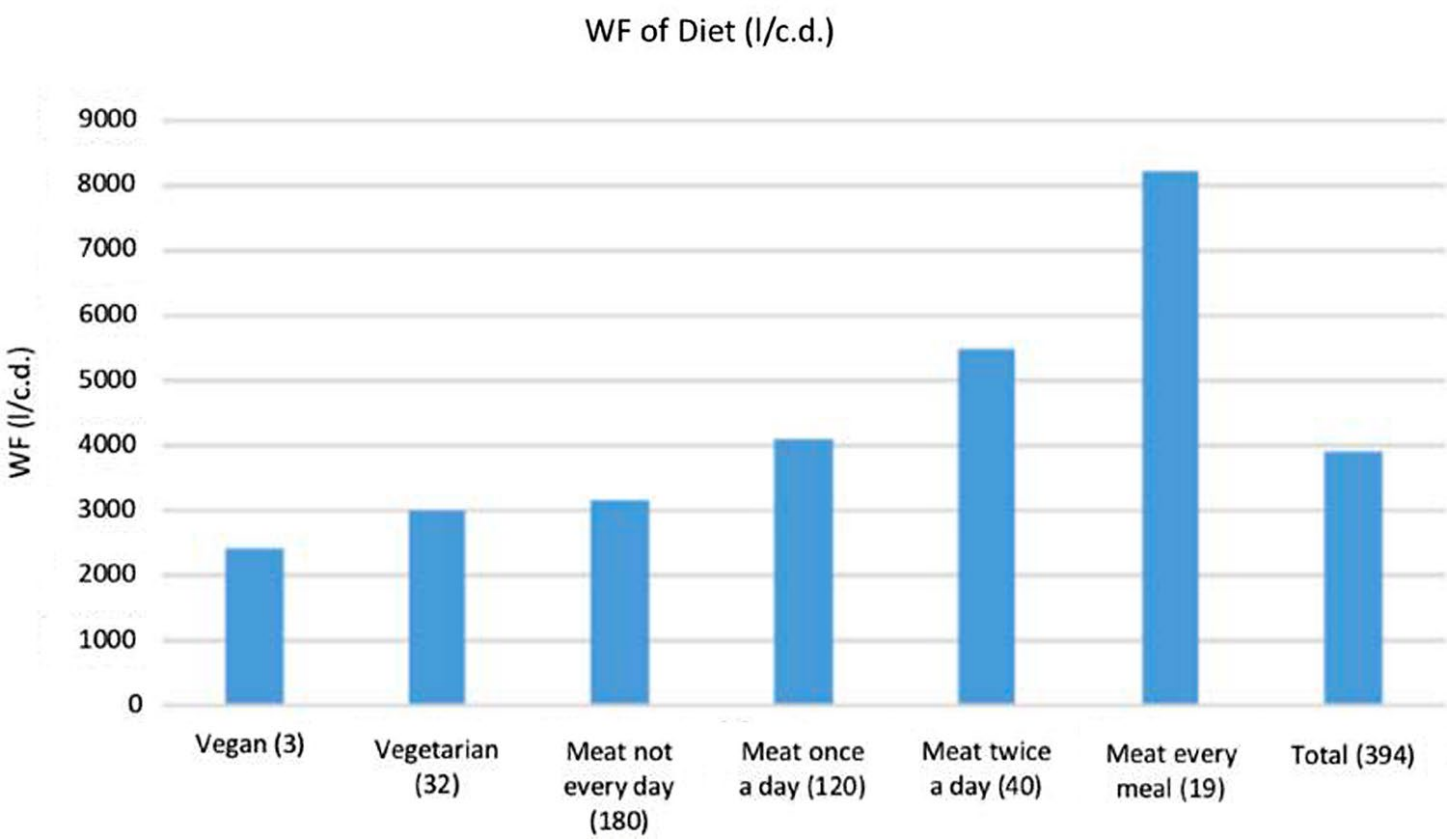
WF (l/c.d.) of diet preferences of BU

The highest WF proportion of students of Boğaziçi University is due to their eating preferences. A total of 394 students responded to the survey of which 3 students are vegan; 32 students are vegetarian; 180 students eat meat, not every day, 120 students eat meat once a day; 40 students eat meat twice a day, and 19 students eat meat every meal of the day. WF of the diet of Boğaziçi University is 3901 liters per capita per day, while that of the USA is 5280 liters per capita per day (Fig. 6).

The second-highest WF proportion of students of Boğaziçi University is due to their shopping preferences. A total of 394 students responded to the survey of which 292 students shop for basics, meaning that they only shop when they need something; 84 students like to shop; 18 students shop whenever they are ready to shop. WF of shopping at Boğaziçi University is 1489 liters per capita per day, while WF of shopping in the USA is 2206 liters per capita per day. The third highest proportion of WF of students of Boğaziçi University is due to cat and dog food consumption. WF of cat and dog food of Boğaziçi University is 240 liters per capita per day, while that of USA is 140 liters per capita per day.

### Some remarks about WF for Boğaziçi University

Not only students of Boğaziçi University but most of the people seem to not understand and underestimate the importance of water. Unnecessary and extreme use of water will cause water shortage in near future. Water is a vital source of the things that people consume and use every day such as food, clothing, gasoline for a car, construction for homes. To reduce water footprint, some suggestions are given below:

- While conducting the survey, it was seen that most of the students do not know the meaning of water footprint. Personal water footprints and the meaning them should be taught and a self-awareness environment should be constructed.
- On average, respondents leave the water running for 9.5 min in the bathroom sink, and 11.6 min in the kitchen sink. Water should be turned off while shampooing, shaving, brushing teeth, and cleaning dishes. In this way, wasting water will be minimized.
- Gardening consumes a great amount of water. Use of less water while gardening, and cover their pools when not using should be tried.
- On average, only 10% of respondents recycle every recyclable material. The habit of recycling and not wasting, going zero-waste, should be gained.
- 30% of the respondents use energy and water-efficient devices. The use of energy and water-efficient (low-flow) devices should be increased.
- 6% of respondents have a greywater system installed at home. Greywater systems should be installed and used to reduce water footprint.
- 5% of respondents have a rain barrel system installed. To save water and decrease water footprint, rain barrel use should be increased.
- 3% of respondents have plants at their homes. Plants decrease evapotranspiration; less water-consuming plants should be planted.
- The water footprint of the eating habits of students of Boğaziçi University is 3901 liters per capita per day; 4% of respondents eat meat in every meal, and 46% of respondents eat meat in more than one meal. Changing eating habits into less water-consuming options such as eating less meat, and drinking less coffee, or switching coffee with tea will decrease water footprint.
- Respondents may use the toilet as a waste bin. The toilet is not a waste bin; waste should be thrown away in waste bins.
- 25% of respondents have a car. Car sharing will allow students who use the same route to travel together.
- Bicycle use should be increased.
- 74% of respondents shop for basics. If possible, buying unnecessary products should be minimized, in this way wasting can be minimized.

## Conclusion

Since climate change is known to be extremely likely due to human activities, population increase and adverse effects of climate change on freshwater resources will create stress on freshwater resources. By calculating the water footprint of a population starting from small scales, and knowing how much water is consumed is the first step of decreasing or adjusting the daily water use. The aforementioned steps should be taken to decrease water footprint and ease the pressure on water resources. To decrease water footprint, the highest constituent of WF such as dietary preferences and shopping preferences should be adjusted to more sustainable preferences. These can be in terms of eating less meat, drinking less water consumptive beverages, eating less water consumptive foods and reusing more, recycling more, and using green energy. Furthermore, green and smart solutions should be taken with the initiatives of the government and its agencies putting people in the center of these solutions because if the end-user does not accept the change and the solution, policies about decreasing water consumption are prone to be inefficient. This study contributes to the literature by calculating virtual and direct water footprints of ordinary campus residents by examining their daily routines and water use behaviors.

For future studies, some recommendations are given below:

- Larger sample size may give different results by reaching out to a diverse group of people.
- Scenario analysis of dietary preferences can be investigated by manipulating into today's preferences. The eating habits of people may change in difficult times such as COVID-19.
- Attributes may also be investigated during the pandemic.

## Survey questions

### Education and accommodation part

- Your Department
- Your degree
- Your grade
- Age
- Your accommodation
- How many people are in your household?

### Indoor water use

- How long is the average shower in your household?
- Do you have low-flow shower heads?
- Do you take baths? If so, how often?
- How long do you leave your bathroom faucets running each day? (include brushing your teeth or shaving.)
- Do your bathroom sinks have low-flow faucets?
- Do you “let it mellow?”(not flushing every time you use toilet) Do you have low-flow toilets?
- How long do you leave the kitchen faucet running each day? (include rinsing food and cleaning but not washing dishes) Does your kitchen sink have a low-flow faucet?
- How often do you wash your dishes?
- How do you wash your dishes?
- How often do you do laundry?
- How do you laundry?
- Do you have a grey-water system installed in your home?

### Outdoor water use

- Do you water a lawn or garden?
- How much do you water lawn or garden? (area-wise)
- Do you landscape with plants that require little or no water? Do you have a rain barrel?
- Do you have a swimming pool?
- If yes, How many month out of the year do you keep it covered? Do you have a car?
- How often do you wash your car?
- How do you wash your car?

### Virtual water use

- How many kilometers do you drive per week?
- Where does your electricity come from?
- How much do you shop?
- Do you recycle PAPER?
- Do you recycle BOTTLES and CANS?
- Do you recycle PLASTIC?
- Do you donate or re-use old clothing, sheets, blankets and towels? What is your diet?
- How often do you eat meat?
- How much money do you spend on dog and cat food each month? (TRY)
